# Coronary Heart Disease and an Incidental Parathyroid Carcinoma

**DOI:** 10.1155/2019/7159395

**Published:** 2019-07-04

**Authors:** M. Stoll, C. A. Nebiker, L. Remonda, R. Grobholz

**Affiliations:** ^1^Institute of Pathology, Cantonal Hospital Aarau, Aarau, Switzerland; ^2^Department Visceral Surgery, Cantonal Hospital Aarau, Aarau, Switzerland; ^3^Department Neuroradiology, Cantonal Hospital Aarau, Aarau, Switzerland

## Abstract

A 71-year-old woman was admitted with angina pectoris. During hospitalization she developed a myocardial infarction (NSTEMI). Laboratory results revealed a mild elevated troponin and an elevated calcium level (3.35 mmol/l). Subsequently, there was a decreased phosphate (0.36 mmol/l [normal 0.81-1.62 mmol/l]) as well as 16-fold elevated serum level of parathyroid hormone (PTH, 1156 ng/l [normal 10-73 ng/l]), indicating a primary hyperparathyroidism. Sonographically a thyroidal node was detected, not clearly demarcated (TIRADS 5). FNA showed a monomorphic, partial follicular cell population with an immunohistochemical positivity for PTH. Magnetic resonance imaging (MRI) showed a 5 cm large tumor at the right caudal pole of the thyroid with compression of the dorsolateral trachea without infiltration. Surgical removal with en bloc resection of the right hemithyroid with parathyroidectomy was performed. Postoperatively the PTH level dropped to 12.1 ng/l. Pathological examination revealed a 5 cm tumor with infiltration of the thyroid and a perineural invasion. The diagnosis of a presymptomatic parathyroid carcinoma could be established. The affirmative histopathological diagnosis of a parathyroid carcinoma can be challenging and is limited to tumors with evidence of invasive growth in adjacent structures such as the thyroid and/or soft tissue, perineural spaces, angioinvasion of capsular and/or extracapsular vessels, and/or documented metastases.

## 1. Introduction

Parathyroid carcinoma (PC) is a very rare malignant neoplasm that occurs sporadically or as part of a genetic syndrome, called hyperparathyroidism-jaw tumor syndrome (HPT-JT). Approximately 1000 cases of PC were reported in the world literature. The incidence is geographically variable with highest rates in Japan and Italy. Histology is the gold standard for a definitive diagnosis. Herein we present a rare incidentally detected case of a PC.

## 2. Case Report

### 2.1. Clinical Findings

A 71-year-old woman was admitted to the Emergency Department by ambulance with a sudden feeling of faintness and a weakness in both legs. In her medical history she had hypertension and osteoporosis without fractures. Due to a coronary heart disease she received a drug eluting stent (DES) in the left anterior descending coronary artery in 2011. On admission laboratory results revealed a mild elevated troponin I (189 ng/l [normal < 45 ng/l]), initially treated conservatively. On day 2 after hospitalization troponin I level reached 7510 ng/l, qualifying for a non-ST-elevated myocardial infarction (NSTEMI). Subsequently, a percutaneous transluminal coronary angioplasty was performed with insertion of a DES into the right coronary artery. She recovered well from this intervention and troponin I levels dropped to normal levels. Subsequently, a treatment with a dual platelet aggregation inhibitor was initiated (100 mg acetylsalicylic acid and 75 mg Clopidogrel). In parallel to the elevated troponin I on admission, an elevated calcium level of 3.35 mmol/l (normal range 2.15-2.55 mmol/l) was found. Subsequently, there was a decreased serum phosphate (0.36 mmol/l [normal 0.81-1.62 mmol/l]) and vitamin D level (47 nmol/l [normal 50-250 nmol/l]) as well as a 16-fold elevated serum level of parathyroid hormone (PTH) (1156 ng/l [normal 18.4-72 ng/l]).

**Figure 1:**
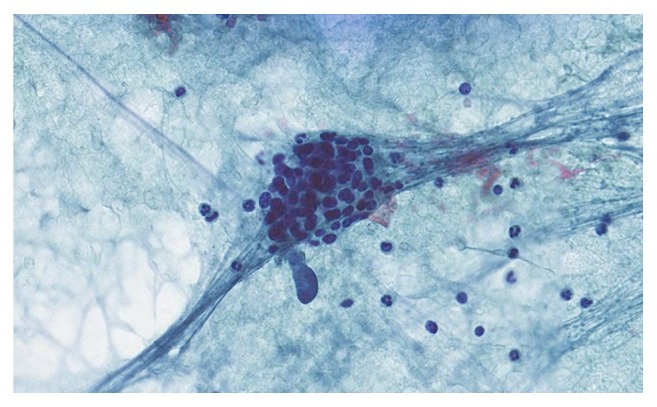
Small monomorphic epithelial cohesive cells and focal follicular arrangements with monomorphic, coarse structured nuclei and a small rim of cytoplasm.

A symptom of the hypercalcemia was polydipsia and consequently the patient also complained about polyuria, but no other clinical manifestation of hypercalcemia such as nephrolithiasis, bone pain, or dyspepsia was noted. On careful clinical evaluation the patient reported a mild dysphagia. A firm and not mobile node on the right side of the neck was palpable. Sonographically a 5 cm right sided thyroidal mass was detected, not clearly definable from the trachea. Using the Thyroid Image Reporting And Data System (TIRADS), the node was categorized as highly suspicious for malignancy (TIRADS 5). Fine Needle Aspiration (FNA) revealed a monomorphic, partial follicular cell population (Figure 1) with focal weak immunohistochemical positivity for PTH. Following these results, a histological clarification was indicated.

**Figure 2:**
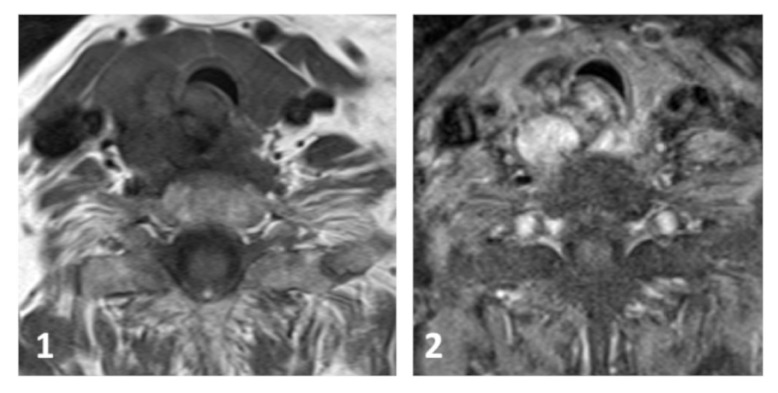
Axial T1-weighted MR image (1) and postcontrast image (2) show a large mass near the right trachea without infiltration.

Computer-assisted tomography (CT) as well as a MRI of the neck revealed a 5 cm large tumor at the right caudal pole of the thyroid with compression of the dorsolateral trachea without obvious infiltration, not distinguishable between thyroid and parathyroid, and tracheal stenosis was about 50% (Figure 2). For a clear distinction between thyroid or parathyroid mass, a parathyroid ^99m^Sestamibi-CT scan was performed. The parathyroid gland takes up radioisotope Technetium-99m Mibi (^99m^Tc) scintigraphy. A second image is obtained after a washout time and mitochondria in the oxyphil cells of the abnormal gland retaining the ^99m^Tc are seen with the gamma camera. The investigation was early stopped, because the patient complained about severe pain. The gained early images showed nodal high uptake of ^99m^Tc Mibi at the right side of the trachea, pointing to a parathyroid origin. Treatment with Cinacalcet 30 mg twice a day was initiated which increases the sensitivity of calcium receptors on parathyroid cells to reduce PTH levels and thus decrease serum calcium levels. Following this treatment calcium levels dropped to nearly normal levels and PTH was still elevated 8 weeks after recovery from the NSTEMI.

### 2.2. Pathologic Findings

A complete surgical removal with en bloc resection of the right hemithyroid with parathyroidectomy was performed. Intraoperatively a large tumor was present, and the recurrent laryngeal nerve could not clearly be identified. Electrostimulation of the assumed nerval structure was unsuccessful. Postoperatively, vocal nerve paresis of the right side was detected in laryngoscopy. After resection PTH values dropped to 90.2 ng/l intraoperatively and postoperatively to 12.1 ng/l. Serum calcium levels normalized to 2.25 mmol/l. While expecting a hungry bone syndrome, a substitution with 1.25-diOH-cholecalciferol and oral calcium was initiated. Without substitution a hypoparathyroidism could lead in the acute phase to tetany with perioral numbness, paresthesia of the extremities, and muscle cramps and in the chronic phase to basal ganglia calcification with movement disorders or dementia, cataracts, or dental defects. Some patients have fewer specific symptoms such as fatigue, hyperirritability, anxiety, or depression and some patients, even with severe hypocalcemia, have no neuromuscular symptoms. Cardiac findings may include a prolonged QT interval, hypotension, heart failure, and arrhythmia. Postoperatively no signs of hypoparathyroidism were obvious, but the patient had dyspnea in context of cardiac insufficiency, averting logopedic voice exercises.

**Figure 3:**
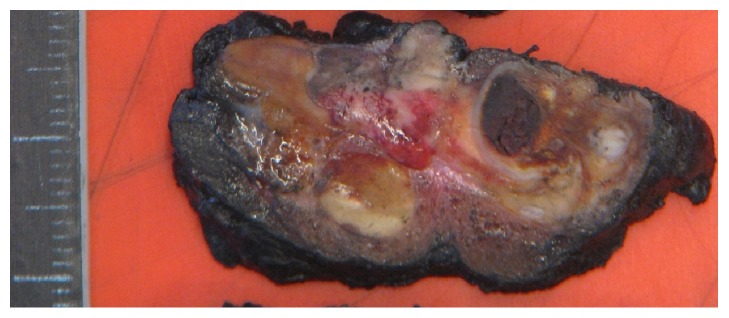
Cut surface of the specimen with a large indistinct colorful tumor with fibrous bands, hemorrhage, and infiltration of the thyroid. Focal perceived brown thyroid on the edge.

**Figure 4:**
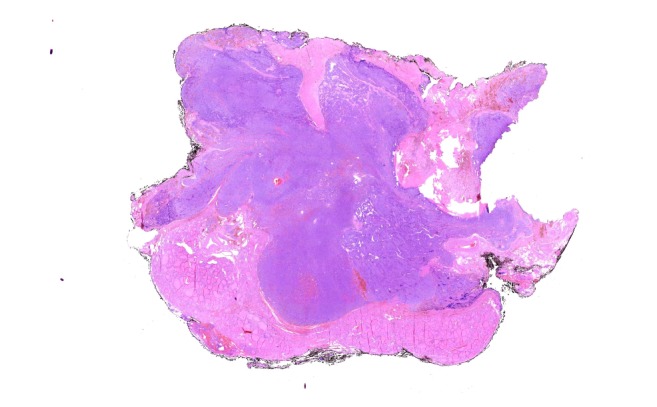
Tongue-like tumor infiltration of the thyroid.

**Figure 5:**
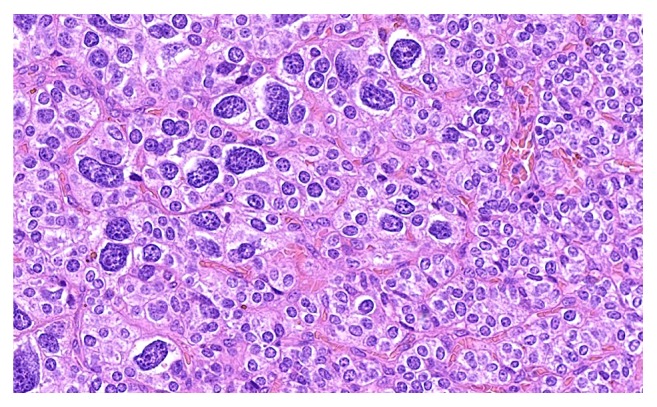
Pleomorphic cells with focal atypia without mitosis.

**Figure 6:**
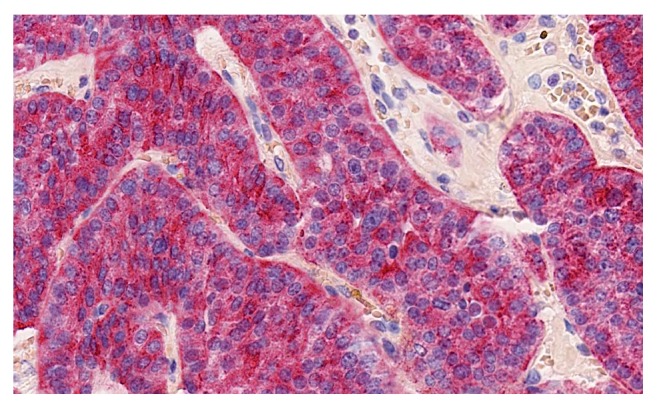
Immunohistochemistry: Cytoplasmic positivity for PTH.

**Figure 7:**
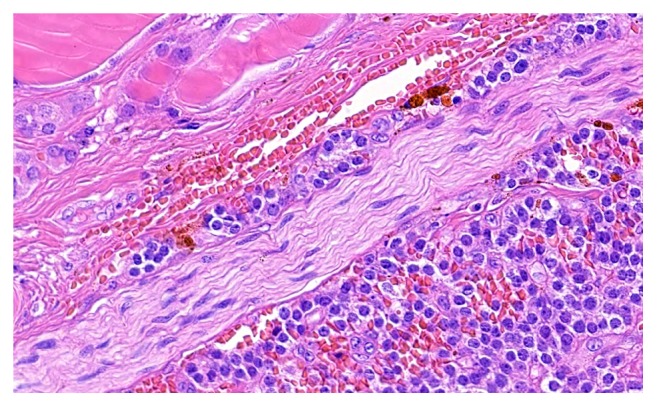
Focal perineural invasion and confirmation of malignancy.

Macroscopic evaluation of the operation specimen revealed a maximal 5 cm large 20g weighing specimen. The cut surface showed a multinodal, partially not clear demarcated protruding greyish-brown firm tumor with hemorrhagic areas, infiltrating the thyroid gland (Figure 3). Microscopic examination showed a parathyroid tumor with multiple fibrous bands dividing the neoplasm into lobules with cellular areas arranged in sheets and trabeculae (Figure 4). The tumor cells were medium to large sized with round to ovoid nuclei containing dense chromatin, inconspicuous nucleoli, and abundant eosinophilic granular to clear cytoplasm. Nuclear pleomorphism was partially present (Figure 5) but no mitotic activity. Immunohistochemically, the tumor showed an expression of PTH (Figure 6) and Galectin-3 and a weak expression of CDKN1B (p27) which is often found in PC [1]. The proliferation rate (as determined by the Ki-67 expression) was 5%. The tumor showed an invasion into the thyroid gland with tongue-like formations and after careful examination, an infiltration of perineural spaces (Figure 7) was found which confirmed the diagnosis of a PC.

## 3. Discussion

PC is an extremely rare malignancy with an annual incidence of approximately 3.5-5.7 per 10 million people [2]. No sex dominance is noted in contrast to benign parathyroid adenomas which show a female to male ratio of 3:1. The mean age of presentation for PC is 56 years and differs significantly from parathyroid adenomas that present 10 years earlier [3]. PC is diagnosed in less than 1% of patients with elevated serum PTH levels [4], and serum levels greater 300 ng/l are highly suggestive for a potentially malignant disease. Clinical signs of hyperparathyroidism typically appear much earlier before a clinically manifest tumor is present. The patients mostly suffer from nephrolithiasis, muscular pain, and dyspepsia [5]. Hypercalcemia and extremely elevated PTH serum levels can be clues to suppose a malignant process. With the exception of patients with germline CDC73 mutation, in whom PC development is consistent with a progression model, allelic loss patterns advocate that most sporadic parathyroid carcinomas arise de novo, rather than evolving from clinically recognizable benign adenomas [6]. PC occurs in 15% of patients with HPT-JT, an autosomal dominant tumor disorder with germline mutations in CDC73. Mostly, parathyroid tumors are the only lesions at presentation, but when additionally jaw tumors are present, the diagnosis of HPT-JT is strongly suggested. Furthermore, there are also benign and a few malignant lesions in other organs: a variety of renal lesions including simple cysts, occasional adenomas hamartomas, and carcinomas [7]. Women have often uterine pathology including leiomyomas, endometrial hyperplasia, polyps, adenomyosis, and adenofibrosis [8]. Another CDC73 related disorder is familial isolated hyperparathyroidism, mostly associated with adenomas of one or more parathyroid glands and sometimes with PC. PC is very rare and not a component of any other heritable syndrome, with the exception of single case reports in the background of MEN1 and MEN2A. Up to date the literature records 17 cases of PC in the setting of MEN1. Studies on PC using next generation sequencing confirmed the importance of CDC73 mutation in the pathogenesis of sporadic and familial PC. Also several interesting candidate oncogenes with lower levels of possible involvement were identified, including PRUNE2, PIK3CA, KMT2D, MTOR, ADCK1, and members of a kinase family with functions related to cell migration and invasion [9, 10]. Until now, we do not have a patient agreement for a molecular analysis in the actual case.

Up to date, histology is the gold standard in diagnosis of a PC. Although fibrosis and mitotic activity are common in PC, these features are not specific for malignancy. Mitotic figures are present in as many as 80% of PC but they can also occur in substantial numbers of parathyroid adenomas. Mitotic activity can be quite low, but most PC have more than 5 mitoses per 50 high power fields [11, 12]. Presence of intratumoral vascular invasion is not considered as a criterion of malignancy [3]. The diagnosis of carcinoma should be restricted to those tumors that show invasion of perineural spaces, soft tissue, thyroid gland, or adjacent structures including blood vessels. Metastases are a sign of malignancy, mostly occurring in neck lymph nodes, lung, liver, and bone. Some parathyroid carcinomas infiltrate the recurrent laryngeal nerve with a subsequent hoarseness already before surgical treatment. Confirmation of this theory is the persistent hoarseness after resection. Surgery is the mainstay of treatment and complete surgical excision at presentation offers the best chance of cure. Patients frequently develop local recurrence, accompanied by profound hypercalcemia. Frozen section of a part of PC is a challenge. Affirmative diagnosis of a PC is only possible with an obvious vascular or perineural invasion or infiltration into adjacent structures. Differential diagnosis is an adenoma or an atypical adenoma of the parathyroid gland. The use of fine needle aspiration in suspected cases of PC is not recommended, because there is a risk of diffusion along the needle tract [13]. In general, immunohistochemistry is not necessary for the diagnosis of a PC.

## 4. Conclusions

The affirmative histopathological diagnosis of a PC can be challenging and is limited to tumors with evidence of invasive growth in adjacent structures such as the thyroid, soft tissue, perineural spaces, and angioinvasion of capsular or extracapsular vessels or documented metastases. This case demonstrates the incidental early finding of a PC on the occasion of a NSTEMI. The early detection of the PC prevented the patient from suffering from further complaints of the primary hyperparathyroidism than the described discrete symptoms.
